# Binding Energies and Optical Properties of Power-Exponential and Modified Gaussian Quantum Dots

**DOI:** 10.3390/molecules29133052

**Published:** 2024-06-27

**Authors:** Ruba Mohammad Alauwaji, Hassen Dakhlaoui, Eman Algraphy, Fatih Ungan, Bryan M. Wong

**Affiliations:** 1 Physics Department, College of Science, Qassim University, Qassim 51452, Saudi Arabia; 2 Physics Department, College of Science of Dammam, Imam Abdulrahman Bin Faisal University, Dammam 34212, Saudi Arabia; 3 Nanomaterials Technology Unit, Basic and Applied Scientific Research Center (BASRC), Physics Department, College of Science of Dammam, Imam Abdulrahman Bin Faisal University, Dammam 34212, Saudi Arabia; 4 Department of Physics, Faculty of Science, Sivas Cumhuriyet University, Sivas 58140, Turkey; 5 Nanophotonics Research and Application Center, Sivas 58070, Turkey; 6 Materials Science & Engineering Program, Department of Chemistry, and Department of Physics & Astronomy, University of California-Riverside, Riverside, CA 92521, USA

**Keywords:** optical absorption coefficient, binding energy, GaAs quantum dot, Schrödinger equation, hydrogenic impurity

## Abstract

We examine the optical and electronic properties of a GaAs spherical quantum dot with a hydrogenic impurity in its center. We study two different confining potentials: (1) a modified Gaussian potential and (2) a power-exponential potential. Using the finite difference method, we solve the radial Schrodinger equation for the 1s and 1p energy levels and their probability densities and subsequently compute the optical absorption coefficient (OAC) for each confining potential using Fermi’s golden rule. We discuss the role of different physical quantities influencing the behavior of the OAC, such as the structural parameters of each potential, the dipole matrix elements, and their energy separation. Our results show that modification of the structural physical parameters of each potential can enable new optoelectronic devices that can leverage inter-sub-band optical transitions.

## 1. Introduction

Quantum structures such as quantum wells, quantum dots (QDs), and nanowires are low-dimensional semiconductors that have enabled several technologies, such as single-electron transistors [1], photovoltaic (PV) devices [2], light-emitting diodes (LEDs) [3], and photodetectors [4,5,6,7,8]. QDs are particularly useful in optoelectronic applications due to quantum confinement effects that enable efficient luminescence, large extinction coefficients, and extensive lifetimes [9,10,11]. For this reason, QDs are presently employed in various applications, including LEDs, photovoltaics, biomedical imaging, solid-state lighting, QD displays, biosensors, and quantum computing materials [12,13,14,15,16,17,18,19]. QDs can be considered a middle ground between molecules and semiconductor materials that enable quantum mechanical properties that can be tailored by varying their physical features [20,21,22,23,24,25,26]. For example, inserting a hydrogenic impurity at the center of a QD center affects the electronic distribution of all energy levels, their separations, and the electronic wavefunctions. This, in turn, affects the electrostatic attraction between the hydrogenic impurity and free carriers, the dipole matrix elements, and the optical absorption coefficient (OAC). There have been several studies that have examined the effects of inserting an impurity in the center of a QD [25,27,28,29,30,31,32,33]. The OACs in coupled InAs/GaAs QD systems were studied by Li and Xia, who found that the optical properties in these QD systems were different from QD superlattices [34]. Schrey and coauthors studied the polarization and optical absorption properties in QD-based photodetectors and found that the QD enables large effects on the distribution of minibands in the superlattice [35]. The variation of the OAC and nonlinear refractive index (NRI) as a function of the applied electric field, temperature, and hydrostatic pressure in a Mathieu-like QD potential with a hydrogenic impurity was examined by Bahar et al. [36]. Batra and coauthors also evaluated the effect of a Kratzer-like radial potential on the OAC and NRI of a spherical QD [37]. Bassani and Buczko studied the sensitivity of the optical properties to the impurity of donors and acceptors in spherical QDs [38]. Narvaez and coauthors examined OACs arising from conduction-to-conduction and valence-to-valence bands [39]. A. Ed-Dahmouny et al. studied the effects of electric and magnetic fields on donor impurity electronic states and OACs in a core/shell GaAs/AlGaAs ellipsoidal QD [40]. In their study, they showed that changes in the polarization of light caused blue or red shifts in the inter-sub-band OAC spectra, depending on the orientations of the two external fields and the presence/absence of a hydrogenic impurity. Fakkahi et al. examined the OACs of spherical QDs based on a Kratzer-like confinement potential [41]. In their study, they demonstrated that the OACs and transition energies (1*p* - 2*s* and 2*s* - 2*p*) were strongly influenced by the structural parameters of the Kratzer confinement potential.

In addition, the oscillator strengths in spherical QDs with a hydrogenic impurity were computed by Yilmaz and Safak [42]. Finally, Kirak et al. studied the effect of an applied electric field on OACs in parabolic QDs with a hydrogenic impurity [43]. In recent years, GaAs-based spherical quantum dots have emerged as a subject of intense research due to their unique properties. GaAs has a high electron mobility, good thermal stability, and excellent optical properties. Moreover, GaAs is widely used in thin film production and high-quality epitaxial growth methods. These factors collectively render GaAs quantum dots appealing for advancing high-performance semiconductor devices and facilitating nanoscale optoelectronic applications. 

In this work, we compute the two lowest energies, $E_{1p}$ and $E_{1s}$, in GaAs spherical quantum dots as a function of the structural shape of two confining potentials: (1) a modified Gaussian potential (MGP) and (2) a power-exponential potential (PEP). We then present a complete analysis of OACs and binding energies as a function of energy separation and dipole matrix elements, as the structural parameters of these potentials are varied. The binding energy effectively captures the attractive force between the free electrons in different levels and the inserted impurity. Section 2 provides the mathematical details of our approach, and Section 3 presents our results for each potential. 

## 2. Theoretical Details

### 2.1. Geometrical Forms of MGP and PEP Potentials

Before calculating the different energy levels and electronic wavefunctions in the QD, we first evaluate the effects of the structural parameters on the geometrical shape of the confining potentials. The spherical symmetry of these potentials introduces a quantization of the angular motion via the angular and magnetic numbers. Within this quantization, the total carrier wavefunction can be expressed by the well-known spherical harmonics. The adjustment and control of electronic transitions in QDs can be attained by varying the size of each layer in the structure or by changing the structural parameters governing the shape of the potentials.

**Figure 1:**
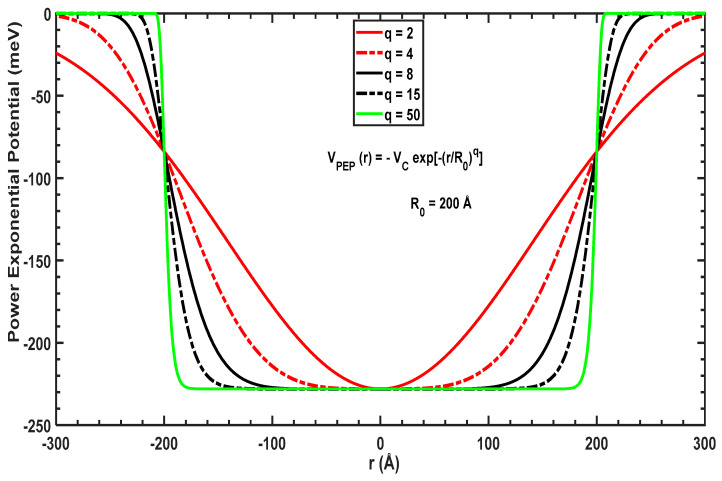
$V_{PEP}\left(r\right)$ for different values of the parameter q with $R_{0}=200\;Å$.

**Figure 2:**
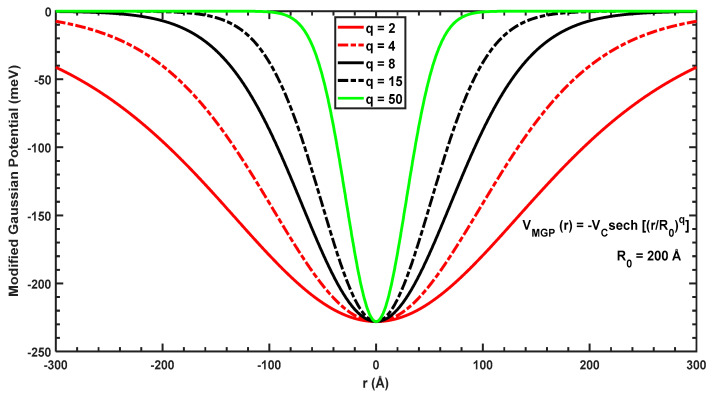
$V_{MGP}\left(r\right)$ for different values of the parameter q with $R_{0}=200\;Å$.

In the present paper, we examine two confining potentials: (1) the power-exponential potential, $V_{PEP}\left(r\right)$, and (2) the modified Gaussian potential, $V_{MGP}\left(r\right)$. These potentials are generated by the application of an external voltage and barriers/wells of the structure with analytical expressions given by the following [44,45,46,47,48]:

(1) $$V_{PEP}\left(r\right)=-V_{c}\;exp\left[-{\left(\frac{r}{R_{0}}\right)}^{q}\right],$$

(2) $$V_{MGP}\left(r\right)=-V_{c}\;sech\left[{\left(\frac{r}{R_{0}}\right)}^{q}\right],$$

where $V_{c}$ and $R_{0}$ are the depth and range, respectively, of these potentials, and q is a structural parameter. Figure 1 and Figure 2 plot the two potentials as a function of the radius for a GaAs QD with different values of the structural parameter, q. Figure 1 shows that $V_{PEP}\left(r\right)$ has a global minimum of $-V_{c}$ at r=0 and increases for higher values of r. For low values of q, the potential has a parabolic shape but gives a square-like confining potential for larger values of q. By increasing q, the potential widens but has the same value at $r=R_{0}$ regardless of the value of q. These geometrical changes enable us to understand their effect on the desired energy levels and optimize the transitions between the initial and final levels to obtain the desired absorption.

Figure 2 plots the modified Gaussian potential as a function of the radius, r. To allow for a straightforward comparison, the same radius of $R_{0}=200\;Å$ is used in Figure 2 (which was considered in Figure 1). When q=2, the power-exponential and modified Gaussian potentials resemble each other; however, when q is increased, the shape of the potential tends to a negative Dirac-delta function at r=0, which will dramatically affect the confinement of wavefunctions and energy levels of the ground and first-excited states.

### 2.2. Optical Absorption of the MGP and PEP Potentials

To compute the $E_{1s}$ and $E_{1p}$ energy levels and the $R_{1s}\left(r\right)$ and $R_{1p}\left(r\right)$ wavefunctions, the radial part of the Schrödinger equation is solved with each of the confining potentials within the effective mass approximation. The Schrödinger equation with the hydrogenic impurity is given by [49,50]:

(3) $$\left[-\frac{\hbar^{2}}{2}\vec{\nabla_{r}}\left(\frac{1}{m^{*}\left(r\right)}\vec{\nabla_{r}}\right)+\frac{l\left(l+1\right)\hbar^{2}}{2m^{*}\left(r\right){\;r}^{2}}-\frac{{Z\;e}^{2}}{\varepsilon \;r}+{\;\;\;V}_{conf}\left(r\right)\right]R_{nl}\left(r\right)={E_{nl}\;R}_{nl}\left(r\right),$$

where ℏ, ε, and l are the reduced Planck constant, dielectric constant, and angular quantum number, respectively. $V_{conf}\left(r\right)$ is the confining potential, $V_{MGP}\left(r\right)$ or $V_{PEP}\left(r\right)$. In addition, $R_{nl}\left(r\right)$ and $E_{nl}$ denote the radial wavefunction and energy level of the confined electron. Including/neglecting the hydrogenic impurity is controlled by setting Z=1 or Z=0, respectively. To find the values of $E_{nl}$ and $R_{nl}\left(r\right)$, the Schrödinger equation is discretized and transformed to an eigenvalue problem, Hx=λx, where H is a tridiagonal matrix, and λ and x represent $E_{nl}$ and $R_{nl}\left(r\right)$, respectively. After discretization, the Schrödinger equation can be written as follows:

(4) $$R_{nl}\left(j+1\right)\left[-\frac{\hbar^{2}}{2m^{*}r_{j}\left(∆r\right)}-\frac{\hbar^{2}}{2m^{*}{\left(∆r\right)}^{2}}\right]+R_{nl}\left(j\right)\left[\frac{\hbar^{2}}{m^{*}{\left(∆r\right)}^{2}}+\frac{l\left(l+1\right)}{m^{*}{\left(r_{j}.∆r\right)}^{2}}+{\;\;\;V}_{conf}\left(j\right)\right]\;+R_{nl}\left(j-1\right)\left[\frac{\hbar^{2}}{2m^{*}r_{j}\left(∆r\right)}-\frac{\hbar^{2}}{2m^{*}{\left(∆r\right)}^{2}}\right]=E_{nl}R_{nl}\left(j\right),$$

where the elements of H are:

(5) $$H_{ij}=\left\{\begin{array}{c} \frac{\hbar^{2}}{m^{*}{\left(∆r\right)}^{2}}+\frac{l\left(l+1\right)}{m^{*}{\left(r_{j}.∆r\right)}^{2}}+V_{KP}\left(j\right),\;\;\;\;\;\mathrm{if}\;j=i\\ \frac{\hbar^{2}}{2m^{*}r_{j}\left(∆r\right)}-\frac{\hbar^{2}}{2m^{*}{\left(∆r\right)}^{2}},\;\;\;\;\;\;\;\;\;\;\;\;\;\;\;\;\;\;\;\;\;\;\;\;\;\;\;\;\mathrm{if}\;j=i-1\\ -\frac{\hbar^{2}}{2m^{*}r\left(∆r\right)}-\frac{\hbar^{2}}{2m^{*}{\left(∆r\right)}^{2}},\;\;\;\;\;\;\;\;\;\;\;\;\;\;\;\;\;\;\;\;\;\;\;\;\mathrm{if}\;j=i+1\\ 0,\;\;\;\;\;\;\;\;\;\;\;\;\;\;\;\;\;\;\;\;\;\;\;\;\;\;\;\;\;\;\;\;\;\;\;\;\;\;\;\;\;\;\;\;\;\;\;\;\;\;\;\;\;\;\;\;\;\;\mathrm{otherwise} \end{array}\right.$$

After discretization, the radial coordinate is $r_{j}=j∆r$ with j=1,…,N, where $∆r=\frac{R}{N}\;$ is the width of the radial mesh. As boundary conditions, the ground and first-excited wavefunctions vanish at the external boundary point (j=N+1) due to the negligible probability of finding the electron at the edge of the confining potential at r=R. In our simulation, we diagonalized the N×N matrix with N=1200 using the MATLAB (version 9.8) software package.

The OACs of different potentials arise from an electronic transition from the 1s to the 1p states after the absorption of a photon having an energy of $\hbar \omega =E_{f}-E_{i}$. We denote OAC as $\alpha \left(\hbar \omega \right)$ and compute it using Fermi’s golden rule with the following expression [51]:

(6) $$\alpha \left(\hbar \omega \right)=\frac{16\pi^{2}\gamma_{FS}N_{if}}{n_{r}V_{con}}\hbar \omega {\left|M_{if}\right|}^{2}\delta \left(E_{f}-E_{i}-\hbar \omega \right).$$

The parameters $\gamma_{FS}$, $V_{con}$, $n_{r}$, and $N_{if}$ are the fine-structure constant, confinement volume, and refractive index, respectively. The Dirac δ-function in Equation (6) can be replaced with the following Lorentzian function [51]:

(7) $$\delta \left(E_{f}-E_{i}-\hbar \omega \right)=\frac{\hbar \Gamma }{\pi \left[{\left(E_{f}-E_{i}-\hbar \omega \right)}^{2}+{\left(\hbar \Gamma \right)}^{2}\right]}.$$

In our study, the initial (i=1) and final (f=2) states are the 1*s* and 1*p* states, respectively. The physical parameters used in this study are: $\gamma_{FS}=1/137$, $n_{r}=3.25$, ℏΓ=3 meV, $m^{*}=0.067\;m_{0}$, and $V_{C}=0.228\;eV$. Furthermore, we use atomic units $\left(\hbar =e=m_{0}=1\right)$ throughout this work, which corresponds to a Rydberg energy and Bohr radius of $1\;R_{y}≅5.6\;meV$ and $1\;a_{B}≅100\;Å$, respectively. In addition, the electromagnetic radiation is polarized along the z-axis, and ${\left|M_{12}\right|}^{2}$ is given by the following expression [51]:

(8) $${\left|M_{12}\right|}^{2}=\frac{1}{3}{\left|\int_{0}^{\infty }R_{1s}\left(r\right)r^{3}R_{1p}\left(r\right)dr\right|}^{2},$$

where the $\frac{1}{3}$ pre-factor arises from the integration of the spherical harmonics.

## 3. Results and Discussion

### 3.1. Optical Properties of GaAs Quantum Dot with PEP Potential

**Figure 3:**
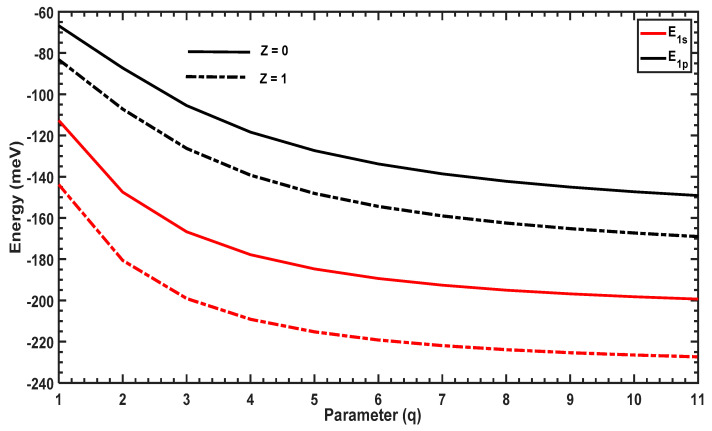
Variations in $E_{1s}$ and $E_{1p}$ as a function of the parameter q. The solid lines are energies without the hydrogenic impurity (Z=0), and the dashed lines represent energies with the hydrogenic impurity (Z=1).

In this section, we will discuss the effect of the structural parameter ,q, on the $E_{1s}$ and $E_{1p}$ energy levels and the binding energy. We then analyze trends in ${\left|M_{if}\right|}^{2}$ and the OACs for the transition between these states. Figure 3 plots the energy levels of the ground (1s) and first-excited (1p) states as a function of the structural parameter q with and without the hydrogenic impurity. When q increases, the energy levels decrease rapidly at low values of q and tend toward constant values, which is due to the shape of the confining potential shown in Figure 1 (the energy levels are inversely proportional to the width of the well). Furthermore, in the presence of the hydrogenic impurity $\left(Z=1\right)$, the energy levels are reduced compared to those in the absence of the impurity $\left(Z=0\right)$ due to the strong attraction between the electrons and the impurity at the center of the QD. In addition, we observe a slow decrease in all energy levels for larger values of q, since the width of the potential (see Figure 1) becomes insensitive to the variation of large q values.

**Figure 4:**
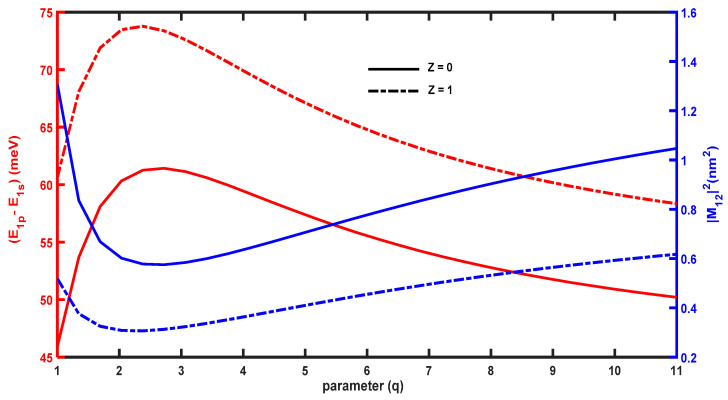
Variations in $E_{1p}-E_{1s}$ and ${\left|M_{12}\right|}^{2}$ as a function of the parameter q.

The OAC between the ground and first-excited levels depends on the energy separation $∆E=E_{1p}-E_{1s}$ and the dipole matrix element, ${\left|M_{12}\right|}^{2}$. Figure 4 plots these physical quantities as a function of the structural parameter, q. For Z=0, ∆E increases, reaches its maximum at q=3, and subsequently decreases. This arises because $E_{1p}$ and $E_{1s}$ decrease when q<3; however, the decrease in $E_{1s}$ is faster than that of $E_{1p}$. As such, ∆E shows an increasing variation; however, the opposite trend occurs for q>3, leading to a reduction in ∆E. Consequently, the OAC can undergo a red or blue shift as q increases. 

Figure 4 shows the variation of the dipole matrix element, ${\left|M_{12}\right|}^{2}$, which plays a crucial role in controlling the amplitude of the optical absorption. ${\left|M_{12}\right|}^{2}$ decreases for q<3 and increases for q>3, which is the opposite trend to that of ∆E. For low values of q, the overlap between the ground and first-excited wavefunctions is reduced; however, the overlap increases for larger values of q, resulting in an enhancement of ${\left|M_{12}\right|}^{2}$.

**Figure 5:**
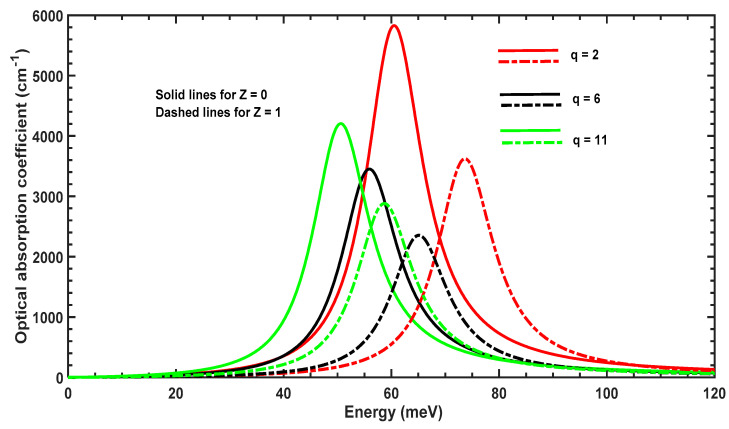
OAC as a function of incident photon energy for different values of the parameter q with $\left(Z=1\right)$ and without $\left(Z=0\right)$ the impurity.

Figure 5 displays the variation of the OAC as a function of the incident photon energy for three values of the parameter q. The OAC peak moves to the left (redshifts) when q is increased, which arises from the variation of the energy separation shown in Figure 4. Furthermore, the amplitude diminishes for q=6 and subsequently rises again when q=11. The amplitude and position of the OAC is sensitive to q, which affects the geometrical shape of the confining potential and delocalization of the 1s and 1p wavefunctions. 

**Figure 6:**
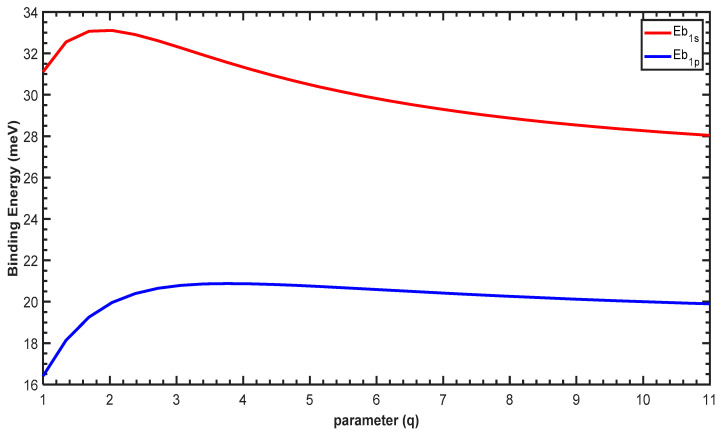
Variation of the binding energy as a function of the parameter q for the 1s and 1p states.

Figure 6 shows the variation of the binding energy (*Eb*) as a function of the parameter q. For low values of q, the binding energy increases sharply for both the 1p and 1s states and subsequently decreases. For all values of q, the binding energy of the 1s state is larger than the 1p state, which is due to the strong electrostatic attraction between the impurity and the electron in the 1s state compared to the 1p state. Furthermore, increasing q enlarges the confining potential, as shown in Figure 1, which leads to a reduction in all energy levels of the QD with and without the impurity. Therefore, the binding energy will be influenced by two effects: (1) the electrostatic attraction and (2) the geometrical confinement imposed by the confining potential. For higher values of q, the confining potential becomes too large and dominates the effect of the electrostatic attraction, leading to a reduction in the binding energies, as shown in Figure 6.

### 3.2. Optical Properties of a GaAs Quantum Dot with an MGP Potential

In this section, we examine the effect of the structural parameter q on the $E_{1s}$ and $E_{1p}$ energy levels, their energy separation, and the binding energy. We then discuss the behavior of the dipole matrix elements and the OACs between these states.

**Figure 7:**
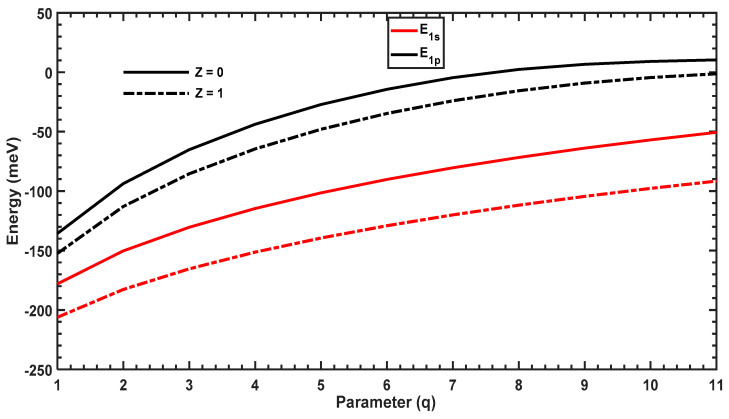
Variation of ${\;E}_{1s}$ and ${\;E}_{1p}$ as a function of the parameter q. The solid lines are energies without the hydrogenic impurity (Z=0), and the dashed lines represent energies with the hydrogenic impurity (Z=1).

Figure 7 plots the energy levels of the ground (1s) and first-excited (1p) states as a function of the structural parameter q with and without the hydrogenic impurity. When q increases, these energies increase considerably in the presence and absence of the impurity, which is opposite to that observed in the previous section for the PEP confining potential. Increasing q reduces the width of the MGP; for higher values of q, the potential tends to the shape of a negative Dirac-delta potential (Figure 2), which increases the energy levels. In addition, the slope of each energy level is slowly reduced for higher values of q since the confining potential no longer changes for very large values of q. 

**Figure 8:**
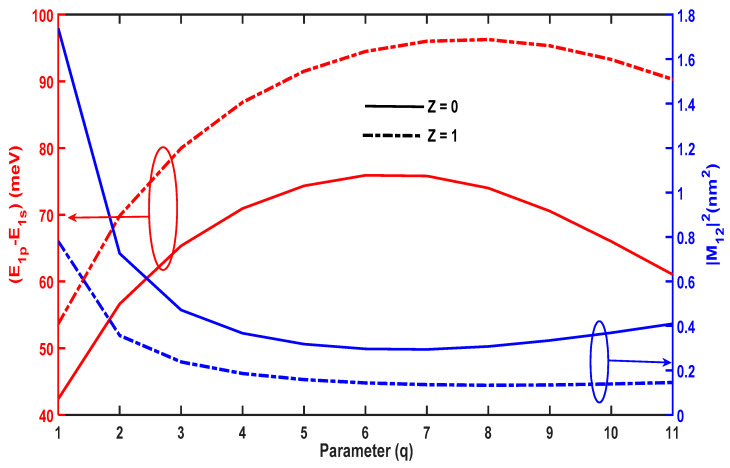
Variations in $E_{1p}-E_{1s}$ and ${\left|M_{12}\right|}^{2}$ as a function of the parameter q.

Comparing Figure 3 and Figure 7, the evolution of the energy levels as a function of the structural parameter are opposite for the PEP and MGP potential. The PEP potential tends to a square-like quantum well, leading to a reduction in energy levels; however, the MGP potential tends to a Dirac-delta form, which shifts all of the energy levels to higher values. Figure 8 plots ${\left|M_{12}\right|}^{2}$ and $∆E=E_{1p}-E_{1s}$ as a function of q, which shows that ∆E increases with q, reaches a maximum, and then diminishes. The maximum of ∆E in the presence of the hydrogenic impurity $\left(Z=1\right)$ is slightly different from that in its absence $\left(Z=0\right)$, which causes the blue and red shifts observed in the OAC. Furthermore, the amplitude of the OAC is sensitive to the variation of the dipole matrix element ${\left|M_{12}\right|}^{2}$. Figure 8 shows that ${\left|M_{12}\right|}^{2}$ first decreases with q, reaches a minimum, and finally increases, which is an opposite trend to that of the energy separation, $∆E=E_{1p}-E_{1s}$.

**Figure 9:**
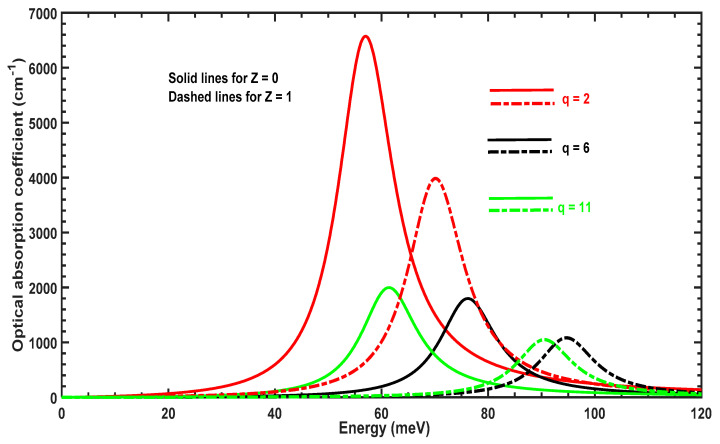
OAC as a function of incident photon energy for different values of parameter *q* with $\left(Z=1\right)$ and without $\left(Z=0\right)$ the impurity.

Figure 9 displays the variation of the OAC as a function of incident photon energy for three values of the parameter q. The OAC peak moves to the right (blue shifts) when q is increased from 2 to 6; it subsequently moves to the left (redshifts) when q increases from 6 to 11. This arises from the variation of the energy separation shown in Figure 8. Furthermore, the amplitude decreases when q varies between 2 and 6 and rises again when q=11. 

**Figure 10:**
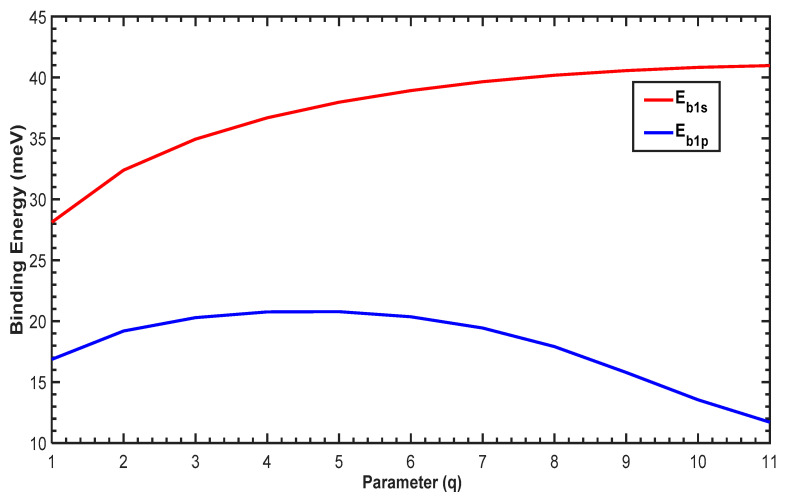
Variation of the binding energy as a function of parameter q for the 1s and 1p states.

Finally, we plot the binding energy in Figure 10. For low values of q, the binding energy increases gradually for the 1p and 1s states. For q>5, the binding energy of the 1p state starts to decrease, whereas the 1s binding energy continues its increase. For all values of the parameter q, the binding energy of the 1s state is larger than that of 1p, which arises from the attraction between the hydrogenic impurity and the free electrons. Furthermore, increasing q subsequently reduces the confining potential (cf. Figure 2), which leads to the enhancement of all energy levels of the QD with and without the presence of the impurity. The difference in the variation of the binding energies in Figure 6 and Figure 10 confirms the effect of the structural parameter q on the PEP and MGP potentials. 

## 4. Conclusions

In this work, we have examined the optical and electronic characteristics of spherical QDs in PEP and MGP potentials. A finite difference method was used to compute the energy levels, OACs, and binding energies for the two low-lying 1s and 1p states. Our calculations for the two confining potentials account for a hydrogenic impurity in the center of the QD. We first calculated the energy levels and their corresponding wavefunctions and subsequently evaluated the dipole matrix elements and energy separations between the 1*s* and 1*p* levels. We then examined the behavior of these physical quantities to interpret the blue and red shifts observed in the variation of OAC.

Our findings show that an increase in the structural parameter of the PEP potential produces a red shift in the OAC, which arises from the change in the energy separation due to the widening of the potential. In addition, our findings showed that an increase in the structural parameter of the MGP potential first produces a blue shift in the OAC and, subsequently, a redshift. The trends in the binding energy as a function of the structural parameter of each confining potential were attributed to the attractive force between the free electrons and hydrogenic impurity. Our simulations provide insight into the optical and electronic characteristics of spherical QDs in various confined potentials.

## Data Availability

The data that support the findings of this study are available from the corresponding author upon reasonable request.
